# Population pharmacokinetics and initial dose optimization of tacrolimus in children with severe combined immunodeficiency undergoing hematopoietic stem cell transplantation

**DOI:** 10.3389/fphar.2022.869939

**Published:** 2022-07-22

**Authors:** Xiao Chen, Dongdong Wang, Feng Zheng, Xiaowen Zhai, Hong Xu, Zhiping Li

**Affiliations:** ^1^ Department of Pharmacy, Children’s Hospital of Fudan University, Shanghai, China; ^2^ Department of Hematology and Oncology, Children’s Hospital of Fudan University, Shanghai, China; ^3^ Department of Nephrology, Children’s Hospital of Fudan University, National Children’s Medical Center, Shanghai, China

**Keywords:** population pharmacokinetics, initial dose optimization, tacrolimus, severe combined immunodeficiency, hematopoietic stem cell transplantation

## Abstract

The present study aimed to explore the population pharmacokinetics and initial dose optimization of tacrolimus in children with severe combined immunodeficiency (SCID) undergoing hematopoietic stem cell transplantation (HSCT). Children with SCID undergoing HSCT treated with tacrolimus were enrolled for analysis. Population pharmacokinetics of tacrolimus was built up by a nonlinear mixed-effects model (NONMEM), and initial dose optimization of tacrolimus was simulated with the Monte Carlo method in children weighing <20 kg at different doses. A total of 18 children with SCID undergoing HSCT were included for analysis, with 130 tacrolimus concentrations. Body weight was included as a covariable in the final model. Tacrolimus CL/F was 0.36–0.26 L/h/kg from body weights of 5–20 kg. Meanwhile, we simulated the tacrolimus concentrations using different body weights (5–20 kg) and different dose regimens (0.1–0.8 mg/kg/day). Finally, the initial dose regimen of 0.6 mg/kg/day tacrolimus was recommended for children with SCID undergoing HSCT whose body weights were 5–20 kg. It was the first time to establish tacrolimus population pharmacokinetics in children with SCID undergoing HSCT; in addition, the initial dose optimization of tacrolimus was recommended.

## Introduction

Severe combined immunodeficiency (SCID), whose estimated incidence of the disease was 1/58,000 (Kwan et al., 2014; Bayram et al., 2021), was an inborn error of immunity characterized by the severe dysfunction of cellular and humoral immunity owing to impaired T cell and B cell development or function (Picard et al., 2018; Miyamoto et al., 2021). This situation caused serious consequences, and affected children, who were born with marked susceptibility to pathogens, could not be managed or controlled at last (Chinn and Shearer, 2015). In addition, without treatment for SCID, infection-related death generally appeared by 1–2 years of age, where these disorders represented true pediatric emergencies (Chinn and Shearer, 2015).

Since 1968, hematopoietic stem cell transplantation (HSCT) had been used to treat patients with SCID (Gatti et al., 1968; Miyamoto et al., 2021). For most forms of SCID, HSCT was the only curative therapy (Bayram et al., 2021). After HSCT, the immune reconstitution and growth were normal in the majority of SCID patients (Demirtas et al., 2021), whose survival was between 85 and 90% in more recent prospective cohorts (Dvorak et al., 2013; Heimall et al., 2017; Haddad and Hoenig, 2019).

For HSCT patients, tacrolimus, an immunosuppressant, needs to be taken for a long time to prevent rejection (Gao and Ma, 2019; Ishiwata et al., 2020; Soskind et al., 2020). However, tacrolimus had high pharmacokinetic variability, making it difficult to formulate an individual administration schedule, especially in children with SCID undergoing HSCT. Thus, the present study aimed to explore the population pharmacokinetics and initial dose optimization of tacrolimus in children with SCID undergoing HSCT.

## Methods

### Patient data collection

Pediatric patients were enrolled from February 2016 to April 2021 at the Children’s Hospital of Fudan University (Shanghai, China), retrospectively. Inclusion criteria were as follows: 1) pediatric patients diagnosed with SCID, 2) pediatric SCID patients underwent HSCT therapy, and 3) HSCT patients treated with tacrolimus. The present study was approved by the Ethics Committee of the Children’s Hospital of Fudan University [Ethical code (2019) 020]. The study was a retrospective analysis, and it was approved by the ethics committee of our hospital without the need for written informed consent. Tacrolimus treatment was performed by clinicians based on the treatment need and clinical experience, and tacrolimus dosage was adjusted based on the clinical efficacy and adverse events experienced by the patients, as well as its trough concentration in therapeutic drug monitoring (TDM). The Emit^®^ 2000 Tacrolimus Assay (Siemens Healthcare Diagnostics Inc., Newark, NJ, United States) with a range of 2.0–30 ng/ml was used to test tacrolimus concentrations. The demographic data of patients and drug combination included gender, age, weight, albumin, alanine transaminase, aspartate transaminase, creatinine, urea, total protein, total bile acid, direct bilirubin, total bilirubin, hematocrit, hemoglobin, mean corpuscular hemoglobin, mean corpuscular hemoglobin concentration, caspofungin, ethambutol, glucocorticoids, isoniazide, micafungin, mycophenolic acid, omeprazole, and vancomycin.

### Population pharmacokinetic model

The population pharmacokinetic model of tacrolimus in pediatric patients with SCID undergoing HSCT was established using the nonlinear, mixed-effects modeling software NONMEM v7 (Icon Development Solutions, Ellicott City, MD, United States) and a first-order conditional estimation method with interaction (FOCE-I) approach. The apparent clearance (CL/F), volume of distribution (V/F), and absorption rate constant (K_a_) were the pharmacokinetic parameters, among which K_a_ was fixed at 4.48/h (Yang et al., 2015; Wang et al., 2019).

### Random-effect model

Eq. (1) was used to estimate the interindividual variabilities,

$$W_{i}=T\left(U\right)\times \exp\left(\eta_{i}\right),$$ (1)

where W_i_ is the individual parameter value. T(U) is a typical individual parameter value. η_i_ is the symmetrical distribution, which was a random term with a zero mean and variance of ω^2^.

Equation (2) was used to estimate the random residual variabilities,

$$M_{i}=N_{i}\times \left(1+\varepsilon_{1}\right)+\varepsilon_{2},$$ (2)

where M_i_ is the observed concentration. N_i_ is the individual predicted concentration. ε_1_ and ε_2_ are the symmetrical distributions, which were random terms with a zero mean and variance of σ^2^.

### Covariate model

Equation (3) was used to estimate the pharmacokinetic parameters and body weight,

$$X_{i}=X_{\mathrm{std}}\times {\left(Y_{i}/Y_{\mathrm{std}}\right)}^{R},$$ (3)

where X_i_ is the i-th individual parameter. X_std_ is a typical parameter. Y_i_ is the i-th individual body weight. Y_std_ is the standard body weight of 70 kg. R is the allometric coefficient: 0.75 for CL/F and 1 for V/F (Anderson and Holford, 2008).

Eqs. (4 and 5) were used to estimate the pharmacokinetic parameters and continuous covariates or categorical covariates,

$$Z_{i}=T\left(Z\right)\times {\left({\mathrm{Co}v_{i}/\mathrm{Cov}}_{\mathrm{median}}\right)}^{\theta },$$ (4)

$$Z_{i}=T\left(Z\right)\times \left(1+\theta \times \mathrm{Co}v_{i}\right),$$ (5)

where Z_i_ is the individual parameter value. T(Z) is a typical individual parameter value. θ is the parameter to be estimated. Cov_i_ is the covariate of the i-th individual. Cov_median_ is the population median for the covariate. The changes in objective function value (OFV) were used as the inclusion criteria for covariates, where the decrease in the OFV > 3.84 (*p* < 0.05) was the inclusion standard, and the increase in the OFV > 6.63 (*p* < 0.01) was the exclusion standard.

### Model evaluation

The goodness-of-fit plots of the model including observations vs. population predictions, observations vs. individual predictions, absolute value of weighted residuals of the individual (│iWRES│) vs. individual predictions, conditional weighted residuals vs. time, the distribution of weighted residuals for the model including density vs. conditional weighted residuals, quantilies of conditional weighted residuals vs. quantilies of normal, the observation/individual predictions/population predictions vs. time, and individual plots were used to estimate the final model. In addition, model stability was evaluated with 1,000 bootstraps with different random sampling.

### Simulation

First, 1,000 virtual pediatric patients with SCID undergoing HSCT were simulated in four body weight groups (5, 10, 15, and 20 kg) with eight dosages (0.1, 0.2, 0.3, 0.4, 0.5, 0.6, 0.7, and 0.8 mg/kg/day), which were divided evenly into two dosages. In addition, Monte Carlo simulations based on the final model were used to study the effects of the initial dosages on the probability of achieving the target concentration (5–20 ng/ml).

## Results

### Patient information

Totally, 18 children (age range: 0.33–3.01 years) with SCID undergoing HSCT were included in the present study. Table 1 showed the demographic data of patients and drug combinations. A total of 130 tacrolimus concentrations were included for analysis, and the mean number of concentrations per patient was 7.2.

**TABLE 1:** Demographic data of patients and drug combination.

Characteristic	Mean ± SD	Median (range)
Gender (boys/girls)	14/4	
Age (years)	0.82 ± 0.56	0.70 (0.33–3.01)
Weight (kg)	7.28 ± 1.62	7.50 (4.20–12.60)
Albumin (g/L)	32.76 ± 3.66	33.20 (25.10–40.80)
Alanine transaminase (IU/L)	38.12 ± 38.05	25.25 (11.00–140.10)
Aspartate transaminase (IU/L)	61.96 ± 31.48	55.60 (29.30–152.00)
Creatinine (μmol/L)	17.72 ± 3.79	18.00 (9.00–27.00)
Urea (mmol/L)	2.77 ± 1.64	2.45 (0.60–7.00)
Total protein (g/L)	57.30 ± 7.35	56.85 (46.80–75.40)
Total bile acid (μmol/L)	7.08 ± 5.33	5.90 (0.10–21.30)
Direct bilirubin (μmol/L)	4.28 ± 5.53	2.40 (0.80–24.40)
Total bilirubin (μmol/L)	8.79 ± 8.53	6.15 (2.20–39.70)
Hematocrit (%)	29.13 ± 7.50	26.61 (22.80–53.31)
Hemoglobin (g/L)	93.58 ± 24.48	87.10 (69.00–167.00)
Mean corpuscular hemoglobin (pg)	25.28 ± 4.16	24.20 (19.00–33.30)
Mean corpuscular hemoglobin concentration (g/L)	321.00 ± 19.56	315.50 (289.00–366.00)
Number of co-medications	-	
Caspofungin	9	
Ethambutol	10	
Glucocorticoids	17	
Isoniazide	14	
Micafungin	9	
Mycophenolic acid	6	
Omeprazole	13	
Vancomycin	10	

### Modeling and evaluation

In the result of the covariate analyze, body weight was included in the final model:

$$\mathrm{CL}/F=13.1\times {\left(\mathrm{WT}/70\right)}^{0.75},$$ (6)

V/F=10900×(WT/70), (7)

where CL/F is apparent clearance. V/F is volume of distribution. WT is body weight.

**FIGURE 1:**
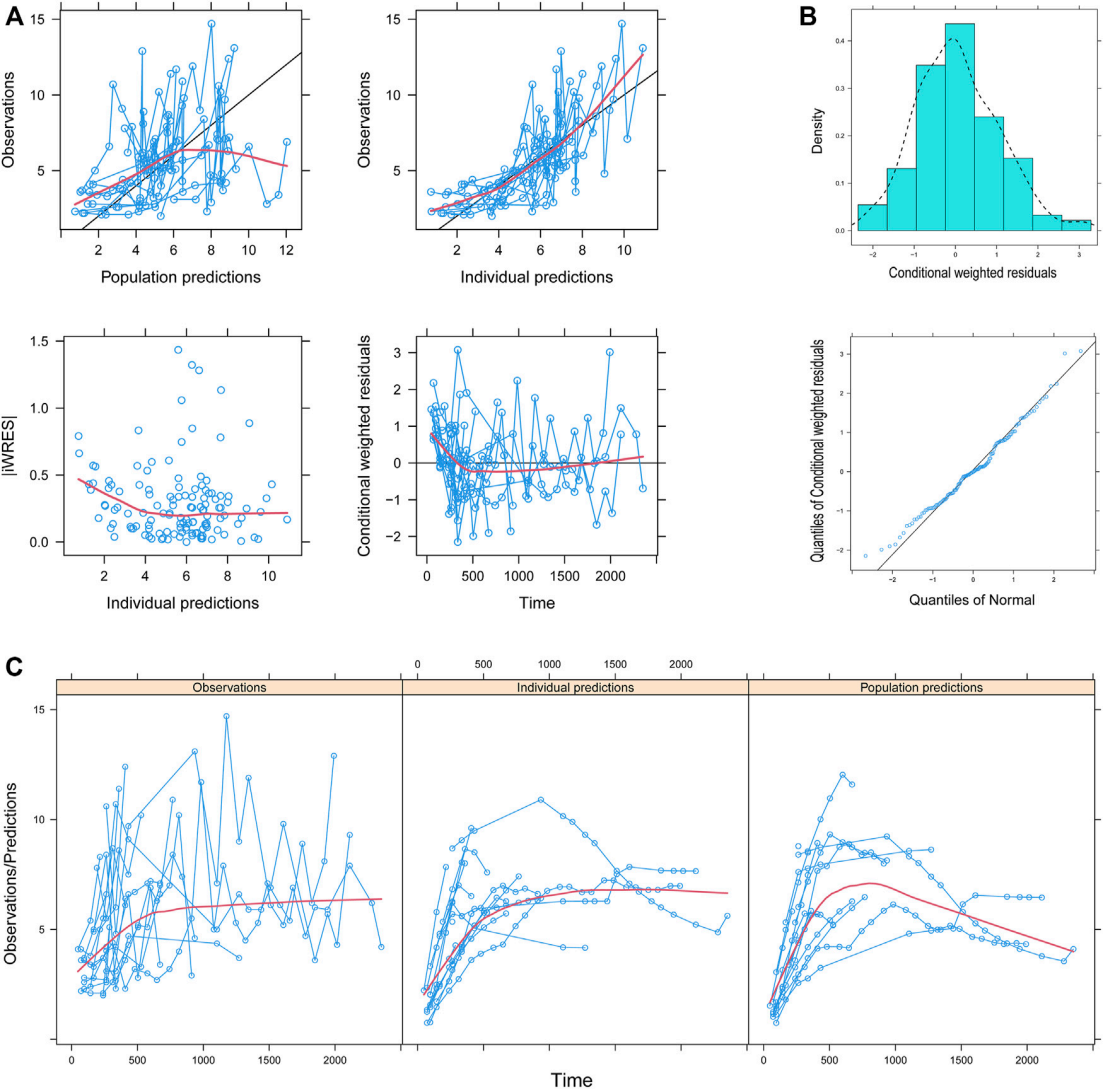
Model evaluation. **(A)** Goodness-of-fit plots of the model, **(B)** distribution of weighted residuals for the model, and **(C)** observation/individual predictions/population predictions vs. time. │iWRES│, the absolute value of weighted residuals of the individual.

**FIGURE 2:**
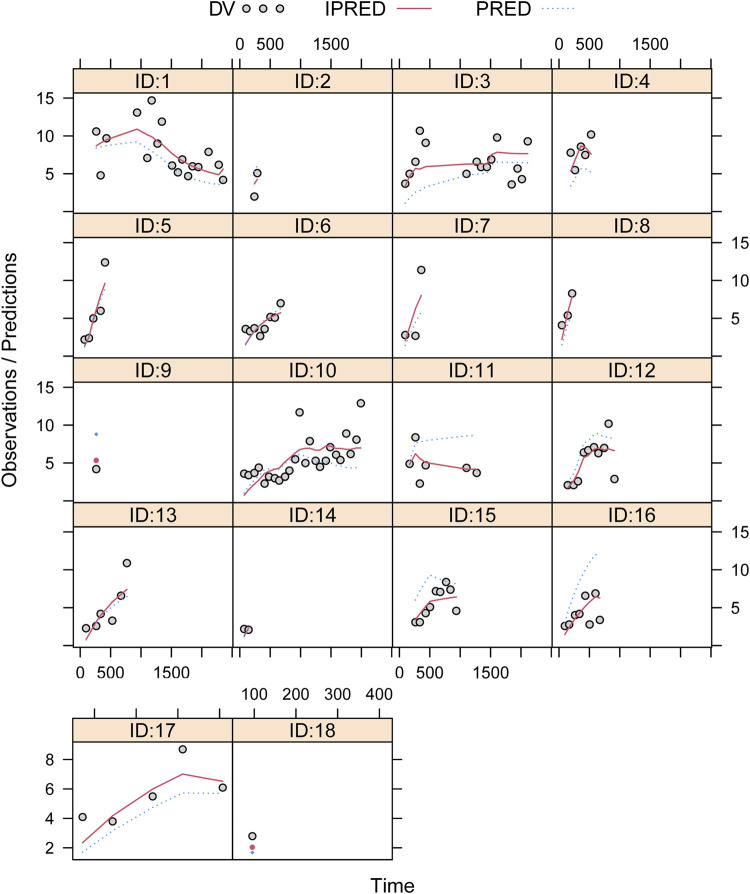
Individual plots. ID, patient ID number; DV, measured concentration value; IPRED, individual predictive value; PRED, population predictive value.

Figure 1 showed the model evaluation. Figures 1A–C were the goodness-of-fit plots of the model, the distribution of weighted residuals for the model, and the observation/individual predictions/population predictions vs. time, respectively. The final model had good performance according to Figures 1A–C. Figure 2 showed the individual plots, demonstrating that the final model had acceptable predictability from a clinical point of view. Table 2 showed the parameter estimates of the final model and bootstrap validation, whose median values of the 1,000 bootstraps were close to the respective parameter values of the final model with a bias <8%, showing that the model was accurate and reliable.

**TABLE 2:** Parameter estimates of final model and bootstrap validation.

Parameter	Estimate	SE (%)	Bootstrap		Bias (%)
			Median	95% confidence interval	
CL/F (L/h)	13.1	27.0	12.8	(8.5, 21.4)	−2.290
V/F (10^2^L)	109	19.5	107	(66, 152)	−1.835
Ka (h^−1^)	4.48 (fixed)	--	--	--	--
ω_CL/F_	0.451	44.8	0.444	(0.003, 0.748)	−1.552
ω_V/F_	0.592	26.4	0.546	(0.003, 0.848)	−7.770
σ_1_	0.257	9.0	0.258	(0.205, 0.336)	0.389
σ_2_	1.265	17.5	1.233	(0.010, 1.587)	−2.530

95% confidential interval was displayed as the 2.5^th^ and 97.5^th^ percentile of bootstrap estimates. CL/F, apparent clearance (L/h); V/F, apparent volume of distribution (L); Ka, absorption rate constant (h^−1^); ω_CL/F_, interindividual variability of CL/F; ω_V/F_, interindividual variability of V/F; σ_1_, residual variability, proportional error; σ_2_, residual variability, additive error; bias, prediction error, bias = (median-estimate)/ estimate×100%.

### Simulation

**FIGURE 3:**
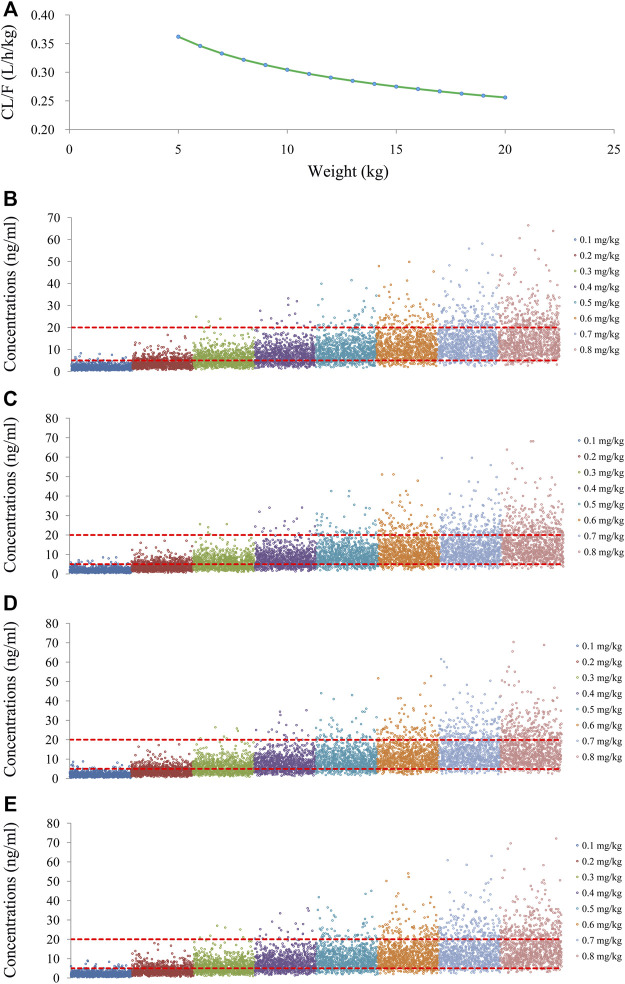
Tacrolimus CL/F and concentration simulation. **(A)** CL/F of tacrolimus in SCID undergoing HSCT. **(B)** Pediatric patients weighing 5 kg. **(C)** Pediatric patients weighing 10 kg. **(D)** Pediatric patients weighing 15 kg. **(E)** Pediatric patients weighing 20 kg.

**FIGURE 4:**
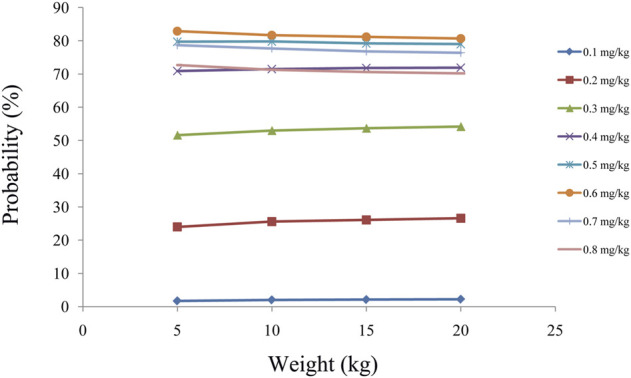
Probability to achieve the target concentrations.

As shown in Figure 3A, tacrolimus CL/F was 0.36–0.26 L/h/kg from body weights of 5–20 kg. We simulated the tacrolimus concentrations using different body weights (5–20 kg) and different dose regimens (0.1–0.8 mg/kg/day). Figures 3B–E showed the results of tacrolimus concentrations for children with SCID undergoing HSCT whose weights were 5, 10, 15, and 20 kg, respectively, where small circles represented drug concentrations, and red dotted lines represented the therapeutic window ranges. Figure 4 showed the probability of achieving the target concentrations under different initial doses of tacrolimus in children with SCID undergoing HSCT, among which the probability of achieving the target concentrations from 0.6 mg/kg/day tacrolimus was the highest. Finally, the initial dose regimen of 0.6 mg/kg/day tacrolimus was recommended for children with SCID undergoing HSCT whose body weights were 5–20 kg.

## Discussion

The clinical manifestation and the treatment outcome of SCID were affected by many factors, such as infectious complications, genetic defects, non-immunological signs, symptoms of the disease, the presence of maternal T cells, and the development of Omenn syndrome (Honig et al., 2011). In terms of treatment, HSCT was the only recognized and reliable therapeutic approach, which allowed long-term cure of the disease (Honig et al., 2011). However, for HSCT patients, tacrolimus, an immunosuppressant, needs to be taken for a long time to prevent rejection (Gao and Ma, 2019; Ishiwata et al., 2020; Soskind et al., 2020).

In clinical practice, tacrolimus required routine TDM to observe the drug concentration of tacrolimus because too low tacrolimus concentration would lead to transplant rejection, while too high tacrolimus concentration would lead to a toxic reaction. This move was necessary because tacrolimus had high pharmacokinetic variability, making it difficult to formulate an individual administration schedule, and the next dose of tacrolimus could only be adjusted through feedback on tacrolimus concentration based on TDM. Although traditional TDM could provide a reference for tacrolimus dose adjustment, it failed when tacrolimus concentration was not available when the first dose needed to be recommended.

Fortunately, the combination of population pharmacokinetics and Monte Carlo simulation could provide a solution to this difficult clinical problem. Importantly, many clinical practices have been carried out and proven to be practical and effective. For example, Cojutti *et al*. reported population pharmacokinetics of continuous infusion of piperacillin/tazobactam in very elderly hospitalized patients and considerations for target attainment against enterobacterales and *pseudomonas aeruginosa* (Cojutti et al., 2021). He *et al*. reported population pharmacokinetics and dosing optimization of vancomycin in infants, children, and adolescents with augmented renal clearance (He et al., 2021). Li *et al*. reported population pharmacokinetics of polymyxin B and dosage optimization in renal transplant patients (Li et al., 2021). Ghoneim et al. (2021) reported optimizing gentamicin dosing in different pediatric age groups using population pharmacokinetics and Monte Carlo simulation. Wang et al. (2021) reported population pharmacokinetics of the anti-PD-1 antibody camrelizumab in patients with multiple tumor types and a model-informed dosing strategy. Yang et al. (2021) reported population pharmacokinetics and safety of dasatinib in Chinese children with core-binding factor acute myeloid leukemia. Chen *et al*. reported population pharmacokinetics and initial dose optimization of sirolimus improving drug blood level for seizure control in pediatric patients with tuberous sclerosis complex (Xiao Chen et al., 2021). Zhang et al. (2020) reported population pharmacokinetics and model-based dosing optimization of teicoplanin in pediatric patients. Based on these precedents, population pharmacokinetics and Monte Carlo simulations were used to recommend optimal initial dosing of tacrolimus in children with SCID undergoing HSCT in our study.

In the previous literature (Wang et al., 2020), pediatric HSCT patients were analyzed as a whole, whereas children with which specific kind of disease undergoing HSCT were not analyzed. However, it was essential to build a specific population pharmacokinetic model of tacrolimus for the specific kind of disease undergoing HSCT (Zhou et al., 2021). Therefore, the present study established tacrolimus population pharmacokinetics in children with SCID undergoing HSCT; in addition, the initial dose optimization of tacrolimus was recommended. In addition, the typical CL/F of tacrolimus in children with SCID undergoing HSCT was 13.1 L/h, and in children with a non-specific kind of disease undergoing HSCT was 15.4 L/h (Wang et al., 2020), hinting that there was a difference from tacrolimus population pharmacokinetics in different kinds of disease undergoing HSCT. In other words, when establishing a population pharmacokinetic model of tacrolimus, the model may be more accurate if the specific kind of disease undergoing HSCT was taken as the population. This was also the necessity for the present study to build the population pharmacokinetics of tacrolimus in children with SCID undergoing HSCT.

In the present study, children with SCID undergoing HSCT treated with tacrolimus were enrolled to analyze, and a total of 18 children with SCID undergoing HSCT were included in the model, with 130 tacrolimus concentrations. Population pharmacokinetics of tacrolimus was built up by a nonlinear mixed-effects model (NONMEM), and initial dose optimization of tacrolimus was simulated using the Monte Carlo method in children weighing <20 kg at different doses. In the final model, body weight was included as a covariable, and tacrolimus CL/F was 0.36–0.26 L/h/kg from body weights of 5–20 kg. Furthermore, we simulated the tacrolimus concentrations using different body weights (5–20 kg) and different dose regimens (0.1–0.8 mg/kg/day). Ultimately, the initial dose regimen of 0.6 mg/kg/day tacrolimus was recommended for children with SCID undergoing HSCT whose body weights were 5–20 kg.

In terms of drug interactions, the present study analyzed caspofungin, ethambutol, glucocorticoids, isoniazide, micafungin, mycophenolic acid, omeprazole, and vancomycin. None of these drugs were found to significantly affect tacrolimus clearance rate as a covariate. Of course, azoles were known to affect the levels of tacrolimus. However, Campagne et al. (2019) reported the model structures of tacrolimus and final covariates mainly depended on the sampling strategy of the study, in other words, the characteristics of the data collected. Numerous covariates were identified as sources of interindividual variability on tacrolimus pharmacokinetics with limited consistency across these studies, which may be the result of the study designs (Campagne et al., 2019). For example, Zhou et al. (2021) reported initial dosage optimization of tacrolimus in pediatric patients with thalassemia major undergoing hematopoietic stem cell transplantation based on population pharmacokinetics, without azoles included as final covariates. Teng et al. (2022) reported population pharmacokinetics of tacrolimus in Chinese adult liver transplant patients, without azoles included as final covariates. Chen *et al*. reported that wuzhi capsule dosage affects tacrolimus elimination in adult kidney transplant recipients, as determined by a population pharmacokinetics analysis, without azoles included as final covariates (Lizhi Chen et al., 2021). Hao et al. (2018) reported population pharmacokinetics of tacrolimus in children with nephrotic syndrome, without azoles included as final covariates. Similarly, due to data limitations, azoles were not analyzed in this study.

Of course, this study also had some limitations. There was a low incidence of SCID in children, leading to our small number of patients for an objective reason. In addition, *CYP3A5* polymorphisms had an effect on tacrolimus metabolism. However, pharmacogenomic consideration in Chinese SCID patients has not been used clinically. The tacrolimus model with polymorphisms might not be practical for simulating drug concentration data from TDM in the real world. Therefore, our model in the present study had better clinical and practical value.

## Conclusion

It was the first time to establish tacrolimus population pharmacokinetics in children with SCID undergoing HSCT; in addition, the initial dose optimization of tacrolimus was recommended. However, due to the low incidence of SCID, it was objectively difficult to collect patients, and the number of patients needs to be further increased in future studies to verify our research results.

## Data Availability

The original contributions presented in the study are included in the article/Supplementary Material; further inquiries can be directed to the corresponding authors.

## References

[B1] Anderson B. J. Holford N. H. (2008). Mechanism-based concepts of size and maturity in pharmacokinetics. Annu. Rev. Pharmacol. Toxicol. 48, 303–332. 10.1146/annurev.pharmtox.48.113006.094708 17914927

[B2] Bayram O. Haskologlu S. Bayrakoglu D. Bal S. K. Islamoglu C. Cipe F. E. (2021). Single-center study of 72 patients with severe combined immunodeficiency: Clinical and laboratory features and outcomes. J. Clin. Immunol. 41 (7), 1563–1573. 10.1007/s10875-021-01062-y 34114123

[B3] Campagne O. Mager D. E. Tornatore K. M. (2019). Population pharmacokinetics of tacrolimus in transplant recipients: What did we learn about sources of interindividual variabilities? J. Clin. Pharmacol. 59 (3), 309–325. 10.1002/jcph.1325 30371942 PMC7395655

[B4] Chinn I. K. Shearer W. T. (2015). Severe combined immunodeficiency disorders. Immunol. Allergy Clin. North Am. 35 (4), 671–694. 10.1016/j.iac.2015.07.002 26454313

[B5] Cojutti P. G. Morandin E. Baraldo M. Pea F. (2021). Population pharmacokinetics of continuous infusion of piperacillin/tazobactam in very elderly hospitalized patients and considerations for target attainment against Enterobacterales and *Pseudomonas aeruginosa* . Int. J. Antimicrob. Agents 58 (4), 106408. 10.1016/j.ijantimicag.2021.106408 34314808

[B6] Demirtas D. Cagdas D. Turul Ozgur T. Kuskonmaz B. Uckan Cetinkaya D. Sanal O. (2021). Long term follow-up of the patients with severe combined immunodeficiency after hematopoietic stem cell transplantation: A single-center study. Immunol. Invest. 51, 739–747. 10.1080/08820139.2020.1869776 33472463

[B7] Dvorak C. C. Cowan M. J. Logan B. R. Notarangelo L. D. Griffith L. M. Puck J. M. (2013). The natural history of children with severe combined immunodeficiency: Baseline features of the first fifty patients of the primary immune deficiency treatment consortium prospective study 6901. J. Clin. Immunol. 33 (7), 1156–1164. 10.1007/s10875-013-9917-y 23818196 PMC3784642

[B8] Gao Y. Ma J. (2019). Tacrolimus in adult hematopoietic stem cell transplantation. Expert Opin. Drug Metab. Toxicol. 15 (10), 803–811. 10.1080/17425255.2019.1675635 31595800

[B9] Gatti R. A. Meuwissen H. J. Allen H. D. Hong R. Good R. A. (1968). Immunological reconstitution of sex-linked lymphopenic immunological deficiency. Lancet 2 (7583), 1366–1369. 10.1016/s0140-6736(68)92673-1 4177932

[B10] Ghoneim R. H. Thabit A. K. Lashkar M. O. Ali A. S. (2021). Optimizing gentamicin dosing in different pediatric age groups using population pharmacokinetics and Monte Carlo simulation. Ital. J. Pediatr. 47 (1), 167. 10.1186/s13052-021-01114-4 34362436 PMC8343923

[B11] Haddad E. Hoenig M. (2019). Hematopoietic stem cell transplantation for severe combined immunodeficiency (SCID). Front. Pediatr. 7, 481. 10.3389/fped.2019.00481 31803700 PMC6877719

[B12] Hao G. X. Huang X. Zhang D. F. Zheng Y. Shi H. Y. Li Y. (2018). Population pharmacokinetics of tacrolimus in children with nephrotic syndrome. Br. J. Clin. Pharmacol. 84 (8), 1748–1756. 10.1111/bcp.13605 29637588 PMC6046506

[B13] He C. Y. Ye P. P. Liu B. Song L. van den Anker J. Zhao W. (2021). Population pharmacokinetics and dosing optimization of vancomycin in infants, children, and adolescents with augmented renal clearance. Antimicrob. Agents Chemother. 65 (10), e0089721. 10.1128/AAC.00897-21 34339268 PMC8448120

[B14] Heimall J. Logan B. R. Cowan M. J. Notarangelo L. D. Griffith L. M. Puck J. M. (2017). Immune reconstitution and survival of 100 SCID patients post-hematopoietic cell transplant: A PIDTC natural history study. Blood 130 (25), 2718–2727. 10.1182/blood-2017-05-781849 29021228 PMC5746165

[B15] Honig M. Schulz A. Friedrich W. (2011). Hematopoietic stem cell transplantation for severe combined immunodeficiency. Klin. Padiatr. 223 (6), 320–325. 10.1055/s-0031-1287826 22052630

[B16] Ishiwata Y. Nagata M. Kiuchi S. Ippongi C. Takeda H. Takahashi H. (2020). Intravenous infusion of fentanyl has No effect on blood concentration of tacrolimus in patients receiving hematopoietic stem cell transplantation. Ther. Drug Monit. 43, 688–691. 10.1097/FTD.0000000000000853 33298744

[B17] Kwan A. Abraham R. S. Currier R. Brower A. Andruszewski K. Abbott J. K. (2014). Newborn screening for severe combined immunodeficiency in 11 screening programs in the United States. JAMA 312 (7), 729–738. 10.1001/jama.2014.9132 25138334 PMC4492158

[B18] Li Y. Deng Y. Zhu Z. Y. Liu Y. P. Xu P. Li X. (2021). Population pharmacokinetics of polymyxin B and dosage optimization in renal transplant patients. Front. Pharmacol. 12, 727170. 10.3389/fphar.2021.727170 34512352 PMC8424097

[B19] Lizhi Chen L. Yang Y. Wang X. Wang C. Lin W. Jiao Z. (2021). Wuzhi capsule dosage affects tacrolimus elimination in adult kidney transplant recipients, as determined by a population pharmacokinetics analysis. Pharmgenomics. Pers. Med. 14, 1093–1106. 10.2147/PGPM.S321997 34511980 PMC8423491

[B20] Miyamoto S. Umeda K. Kurata M. Nishimura A. Yanagimachi M. Ishimura M. (2021). Hematopoietic cell transplantation for severe combined immunodeficiency patients: A Japanese retrospective study. J. Clin. Immunol. 41, 1865–1877. 10.1007/s10875-021-01112-5 34448087 PMC8390179

[B21] Picard C. Bobby Gaspar H. Al-Herz W. Bousfiha A. Casanova J. L. Chatila T. (2018). International union of immunological societies: 2017 primary immunodeficiency diseases committee report on inborn errors of immunity. J. Clin. Immunol. 38 (1), 96–128. 10.1007/s10875-017-0464-9 29226302 PMC5742601

[B22] Soskind R. Xiang E. Lewis T. Al-Homsi A. S. Papadopoulos J. Cirrone F. (2020). Initial tacrolimus weight-based dosing strategy in allogeneic hematopoietic stem-cell transplantation. J. Oncol. Pharm. Pract. 27(6), 1447–1453. 10.1177/1078155220959416 32957861

[B23] Teng F. Zhang W. Wang W. Chen J. Liu S. Li M. (2022). Population pharmacokinetics of tacrolimus in Chinese adult liver transplant patients. Biopharm. Drug Dispos. 43 (2), 76–85. 10.1002/bdd.2311 35220592

[B24] Wang D. D. Chen X. Fu M. Zheng Q. S. Xu H. Li Z. P. (2019). Model extrapolation to a real-world dataset: Evaluation of tacrolimus population pharmacokinetics and drug interaction in pediatric liver transplantation patients. Xenobiotica. 50, 371–379. 10.1080/00498254.2019.1631505 31192749

[B25] Wang D. Chen X. Xu H. Li Z. (2020). Population pharmacokinetics and dosing regimen optimization of tacrolimus in Chinese pediatric hematopoietic stem cell transplantation patients. Xenobiotica. 50 (2), 178–185. 10.1080/00498254.2019.1601791 30938547

[B26] Wang C. Y. Sheng C. C. Ma G. L. Xu D. Liu X. Q. Wang Y. Y. (2021). Population pharmacokinetics of the anti-PD-1 antibody camrelizumab in patients with multiple tumor types and model-informed dosing strategy. Acta Pharmacol. Sin. 42 (8), 1368–1375. 10.1038/s41401-020-00550-y 33154554 PMC8285417

[B27] Xiao Chen X. Wang D. Zhu L. Lu J. Huang Y. Wang G. (2021). Population pharmacokinetics and initial dose optimization of sirolimus improving drug blood level for seizure control in pediatric patients with tuberous sclerosis complex. Front. Pharmacol. 12, 647232. 10.3389/fphar.2021.647232 33995061 PMC8114543

[B28] Yang J. W. Liao S. S. Zhu L. Q. Zhao Y. Zhang Y. Sun X. Y. (2015). Population pharmacokinetic analysis of tacrolimus early after Chinese pediatric liver transplantation. Int. J. Clin. Pharmacol. Ther. 53 (1), 75–83. 10.5414/CP202189 25207550

[B29] Yang F. Zhang L. Zhao B. B. Zhang J. L. Liu X. T. Li X. (2021). Population pharmacokinetics and safety of dasatinib in Chinese children with core-binding factor Acute myeloid leukemia. Clin. Pharmacokinet. 61, 71–81. 10.1007/s40262-021-01054-6 34240339

[B30] Zhang T. Sun D. Shu Z. Duan Z. Liu Y. Du Q. (2020). Population pharmacokinetics and model-based dosing optimization of teicoplanin in pediatric patients. Front. Pharmacol. 11, 594562. 10.3389/fphar.2020.594562 33363469 PMC7753357

[B31] Zhou S. Zhang R. Lv C. Lu J. Wei Y. Li C. (2021). Initial dosage optimization of tacrolimus in pediatric patients with thalassemia major undergoing hematopoietic stem cell transplantation based on population pharmacokinetics. Ann. Pharmacother. 55 (4), 440–451. 10.1177/1060028020959039 32924532

